# Late Pleistocene climate change promoted divergence between *Picea asperata* and *P. crassifolia* on the Qinghai–Tibet Plateau through recent bottlenecks

**DOI:** 10.1002/ece3.2230

**Published:** 2016-06-07

**Authors:** Hao Bi, Wei Yue, Xi Wang, Jiabin Zou, Lili Li, Jianquan Liu, Yongshuai Sun

**Affiliations:** ^1^ MOE Key Laboratory for Bio‐resources and Eco‐environmentCollege of Life SciencesSichuan UniversityChengdu610065China; ^2^ State Key Laboratory of Grassland Agro‐ecosystemCollege of Life SciencesLanzhou UniversityLanzhou730000China; ^3^ Key Laboratory of Tropical Forest EcologyXishuangbanna Tropical Botanical GardenChinese Academy of SciencesMengla666303China

**Keywords:** Bottlenecks, *Picea*, Pleistocene climate change, Qinghai–Tibet Plateau, species divergence

## Abstract

Divergence during the early stage of speciation can be driven by a population bottleneck via reduced gene flow and enhanced lineage sorting. In this study, we aimed to examine whether such bottlenecks occurred during the initial speciation of two closely related spruce species *Picea asperata* and *P. crassifolia* occurring on the Qinghai–Tibet Plateau (QTP). We analyzed sequences of three chloroplast, two mitochondrial DNA fragments and a further 13 nuclear loci from 216 individuals of the two species. Both species showed a low level of genetic diversity in contrast to other congeners occurring in the QTP and adjacent regions. The estimated population sizes of *P. asperata* and *P. crassifolia* are less than the ancestral population size before splitting. These results together with multiple statistical tests (Tajima's *D*, Fu and Li's D* and F*) suggest that these two species underwent recent bottlenecks. Based on approximate Bayesian computation (ABC), we also determined that the period of the population shrinkage was consistent with the interspecific divergence during the late Pleistocene. The reduced population sizes and the divergent selection may together have triggered the initial divergence under high gene flow between these two species. Our results therefore highlight the importance of climatic oscillations during the late Pleistocene in promoting speciation through changing demographic sizes of the ancestral species on the QTP and in adjacent regions.

## Introduction

Divergence can be promoted by population bottlenecks because of reduced quantity of gene exchange and that small populations with increased rates of genetic drift can fix more derived mutations than large populations (Templeton 1980, 2008; Barton and Charlesworth 1984). Recently, many studies have focused on the earlier stage of speciation (Abbott et al. 2013; Liu et al. 2014), and some of them have shown that divergence among species can occur even in the presence of gene flow and that species boundaries are maintained by divergent selection (Hewitt 1996; Petit and Excoffier 2009; Schluter 2009; Nosil 2012; Sousa and Hey 2013). However, whether population bottlenecks have played a role in promoting divergence between closely related taxa has rarely been studied (Butlin et al. 2012).

When the strength of random genetic drift increases following reduced population size during a bottleneck period or during the colonization of a new environment, most of the genetic variation within a species is likely to be lost (Young et al. 1996; Excoffier et al. 2009). In addition, when the population size decreases below a critical level, effective mating with conspecifics tends to be difficult to maintain (May and McLean 2007). Populations during a bottleneck period are always at a high risk of extinction due to the lack of adapted variations or mating partners (May and McLean 2007; Excoffier et al. 2009). The lack of genetic variations makes it difficult, experimentally, to model and examine historical bottlenecks of target species (Hein et al. 2004). Therefore, it is challenging to find empirical evidence for the hypotheses that historical bottlenecks promoted or accompanied the initial speciation stage. However, a high level of genetic variation is frequently found in woody species pollinated over long distances by the wind, for example, spruces (Heuertz et al. 2006; Li et al. 2015; Sun et al. 2015). Thus, enough but fluctuated levels of genetic variation in these species may allow us to detect the signals of bottlenecks that occurred during the evolutionary divergence of closely related species. In this study, we aimed to examine whether bottlenecks promoted divergence between *Picea asperata* Masters and *P. crassifolia* Komarov.

**Figure 1:**
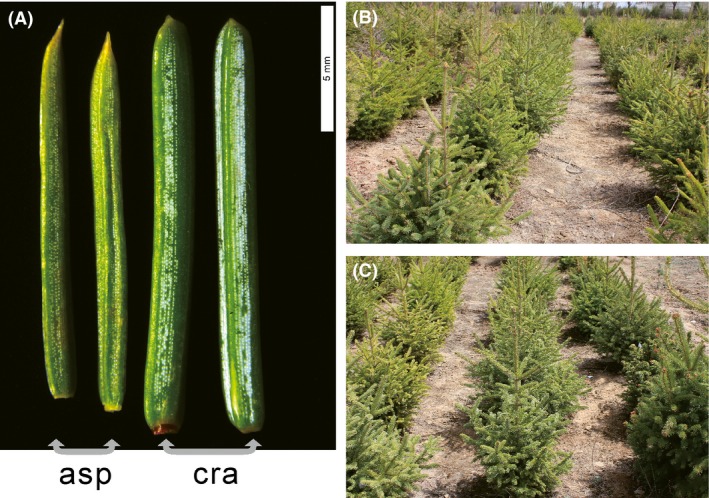
(A) Needles collected from 5‐year‐old trees in a common garden. The left two needles were collected from two saplings of *P. asperata* (asp), and the right two needles were from two saplings of *P. crassifolia* (cra). (B, C) The saplings of *P. asperata* and *P. crassifolia* in a common garden planted in 2007–2009.

*Picea asperata* and *P. crassifolia* have wide distributions across the eastern Qinghai–Tibet Plateau (QTP), but *P. crassifolia* extends to a more northerly latitude. Phenotypic differences between *P. asperata* and *P. crassifolia* are obvious. The leaf apices of *P. asperata* are always acute; in contrast, the leaf apex of *P. crassifolia* is obtuse and the length/width ratio of the leaf is always lower than that of *P. asperata* (Fig. 1). The winter buds of *P. asperata* are resinous, unlike those of *P. crassifolia* (Fu et al. 1999). Previous work based on chloroplast and mitochondrial DNA variations showed that these two species were closely related to each other and none of the derived mutations were species specific (Du et al. 2009), indicating that they may have diverged very recently. We used sequence data from 13 nuclear loci and three chloroplast and two mitochondrial DNA fragments to investigate the evolutionary histories of *P. asperata* and *P. crassifolia*, in combination with planting experiments in common garden. We addressed the following questions. Did historical population bottlenecks and gene flow occur when they diverged? If population shrinkages did occur, when did these bottlenecks take place? Were they correlated with the late Pleistocene climate oscillations?

## Materials and Methods

### Sampling, planting, and DNA sequencing

We sampled needles from 11 and 15 populations, respectively, through the main natural distribution range of *Picea asperata* Masters and *P. crassifolia* Komarov between 2007 and 2012 (Fig. S1; Table 1). The latitude, longitude, and altitude of each sampling location were recorded using an eTrex GIS monitor (Germany); detailed information is presented in Table 1. About 3~11 individuals were sampled from each population. The distance between sample trees was at least 100 m. Needles were collected in the field and dried immediately with silica gel. We compared the width of needles with length of ~1.7 cm, and five needles for each of ten trees per species were measured. The significance level of differences was calculated using R version 3.1.1 (two‐sample Student's *t*‐test, https://www.r-project.org/). Seeds collected from 20 trees (10 trees per species from different populations) were planted in a common garden at Lanzhou University (Xiaguanying), in order to observe whether their morphological traits were stable under common environmental conditions.

**Table 1:** List of the sampled individuals of *Picea asperata and P. crassifolia* with their locations and altitudes

Species	Code	Population	Location	Latitude	Longitude	Altitude (m)	Number
*P. asperata*	18	1912	Songpan, SC	32°23′22.98″	103°31′26.28″	2990	11
	19	1917	Lixian, SC	31°31′4.8″	102°55′27″	2410	3
	20	2027	Luhuo, SC	31°9′56.82″	105°52′46.74″	3120	7
	21	2040	Daofu, SC	30°49′55.02″	101°16′46.98″	3510	6
	24	Liu05027	Mianning, SC	28°52′6.3″	102°17′43.92″	2450	11
	25	Liujq‐09xz‐lzt‐119	Zhuoni, GS	34°34′24.6″	103°22′11.5″	2751	11
	26	Liujq‐09xz‐lzt‐140	Ruo'ergai, SC	33°35′32.7″	103°9′9.2″	3408	8
	27	Liujq‐09xz‐lzt‐147	Aba, SC	32°39′2.2″	101°34′57.0″	3201	10
	29	1889	Songpan, SC	32°47′13.26″	103°33′34.68″	3320	9
	30	1962	Heishui, SC	32°6′1.86″	102°56′31.98″	2827	6
	31	2711	Jiuzhaigou, SC	32°52′44.16″	103°39′28.68″	3150	6
*P. crassifolia*	32	ID	Xiahe, GS	35°7′56.88″	102°50′27″	2840	8
	33	1588	Dulan, GS	36°20′23.22″	98°14′52.2″	3700	9
	34	IC	Zhuoni, GS	34°34′38.22″	103°30′3.18″	2900	5
	35	IE	Kangle, GS	34°55′48.24″	103°43′59.58″	2840	9
	36	IE‐1606‐1322	Menyuan, QH	37°10′20.22″	102°9′3.18″	2600	10
	37	718	Qilian, QH	38°9′8.52″	100°16′30.78″	2750	7
	38	XH	Xinghai, QH	35°32′22.56″	99°50′59.76″	3510	10
	39	IE‐1606‐1606	Huangyuan, QH	36°37′51.06″	101°1′37.8″	3520	7
	40	822	Helanshan, NX	38°39′54.42″	105°53′20.46″	2230	11
	41	828	Daluoshan, NX	37°16′47.4″	106°16′59.7″	2390	10
	42	O610101	Jingtai, GS	34°8′2.28″	103°44′30.06″	2660	8
	43	1702	Tongde, QH	34°47′11.7″	100°48′43.26″	3420	8
	44	1450	Tianzhu, QH	36°53′9.66″	101°41′16.14″	2450	11
	45	818	Maduo, QH	34°55′52.26″	98°31′26.76″	4260	6
	46	Qinghaiyunshan1	Huhehaote, IM	40°54′7.74″	111°9′8.52″	2120	9

SC, Yunnan; GS, Gansu; QH, Qinghai; NX, Ningxia; IM, Inner Mongolia.

A total of 13 nuclear loci (4CL, EBS, FT3, GI, M002, M007D1, PCH, Sb16, Sb29, Sb62, se1364, se1390, and xy1420) were sequenced using an ABI 3130xl Genetic Analyzer (Applied Biosystems, Foster City, CA) following the methods in Li et al. (2010). Sequence data were base‐called using PHRED with CodonCode Aligner software (CodonCode Corporation) and checked using MEGA version 5.0 (Kumar et al. 2008). Sequences were aligned by CLUSTAL X (Thompson et al. 1997) with MEGA5. Sequences with heterozygous sites were rephased and separated into two allelic sequences by PHASE (Stephens et al. 2001; Stephens and Donnelly 2003) in the software package DnaSP V5 (Librado and Rozas 2009) with default parameters. All sequences have been deposited in GenBank (Accession Numbers: KX212991‐KX213062). Chloroplast and mitochondrial DNA variation data (*trn*L‐*trn*F, *trn*S‐*trn*G, *ndh*K/C, *nad*1 intron b/c, and *nad*5 intron1) were obtained from previous studies (Meng et al. 2007; Du et al. 2009).

### Analyses of population genetic diversity and structure

Population genetic parameters, including the number of segregating sites (*S*), Watterson's parameter *θ*_*w*_ (Watterson 1975), and nucleotide diversity *π* (Tajima 1983), were computed using DnaSP V5.10.01 (Librado and Rozas 2009), after excluding insertions/deletions (Table S2). For each locus, Tajima's *D* statistic (Tajima 1989), Fu and Li's D* and F* (Fu and Li 1993), and the number of segregating sites were also calculated using DnaSP V5.10.01 (Librado and Rozas 2009). Sequences of *P. breweriana* were downloaded from GenBank and were used as an out‐group when performing D* and F* tests.

To understand interspecific genetic differentiation at neutral nuclear loci, we computed *Φ*_ST_ using Arlequin V3 (Excoffier et al. 2005) and the significance level was based on 1000 permutations. To check whether data from these two species could be delimitated into two groups, NETWORK version 4.6.1.3 was used to determine genealogical relationships among the mtDNA, cpDNA, and nuclear haplotypes at each locus (Bandelt et al. 1999). Indels at each locus were coded as characters using simple indel coding as implemented in SeqState (Muller 2006). In addition, we used the Bayesian algorithm implemented in STRUCTURE ver. 2.3.3 (Pritchard et al. 2000) to infer the possible genetic structure with the admixture model. All 26 populations and 13 loci were used in STRUCTURE analysis. Sites that showed significant statistical association after Bonferroni correction were removed. Linkage disequilibrium among SNPs within genes was limited as the number of SNPs per gene was small. For each *K*‐value (1 ≤ *K *≤* *10), we performed 20 independent runs with a burn‐in of 200,000 and 500,000 iterations, respectively. The program Distruct version 1.1 (Rosenberg 2004) was used to generate graphical representations of the data. The most likely number of clusters was estimated using the original method from Pritchard et al. (2000) and with the *ΔK* statistics (Evanno et al. 2005).

### Analyses of the isolation and migration (IM) model and bottleneck model

We estimated current and ancient population size, divergence time, and interspecific migration rate under the isolation and migration model using an approximate Bayesian computation (ABC) approach implemented in ABCtoolbox (Wegmann et al. 2010). This divergence model under gene flow assumed that an ancestral lineage with population size *N* diverged into two lineages T generations ago. The population sizes of *P. crassifolia* and *P. asperata* are referred to as *N*c and *N*a. The migration rate from *P. crassifolia* to *P. asperata* is referred to as *M*cra‐>*M*asp and that in the reverse direction as *M*asp‐>*M*cra. We used 13 statistics to summarize the pattern of molecular variation at all nuclear loci, including the number of haplotypes within each species, the number of polymorphic sites within each species, the number of private polymorphic sites for each species, the number of pairwise differences, Tajima's *D* (Tajima 1989), Fu's *F*_S_ (Fu 1997), pairwise *F*_ST_, the number of pairwise differences between species, and the total number of polymorphic sites. These statistics were computed by Arlequin version 3.0 for both observed and simulated datasets (Excoffier et al. 2005). Simulated datasets were generated using a standard algorithm in the ABCtoolbox software package, and fastsimcoal was used to perform the simulation (Excoffier and Foll 2011). The gene information is listed in Table S1. For the simulations, the mutation rate was assumed to be 1 × 10^‐8^ per site per generation (Li et al. 2010; Sun et al. 2014). The prior of each parameter was assumed to follow the uniform distribution. A total of 500,000 datasets were simulated by fastsimcoal, and 5000 of them were retained. We applied the regression adjustment general linear model to compute marginal density and to generate posterior distributions of all parameters (Wegmann et al. 2010).

The low genetic diversity in these two species and positive *D*, D* and F* values at many loci (Table 2) along with the estimated population parameters indicated that a population bottleneck could have occurred during the initial divergence of these two species. So, we dated the population bottlenecks for *P. crassifolia* and *P. asperata* using ABCtoolbox. In addition, due to the low differentiation between these two species, we also analyzed the pooled sequence data from the two species to infer the likely historical population bottleneck. The bottleneck model assumed that an ancestral population with population size *N* underwent population contraction *T*b generations ago, then began to expand *T*e generations ago. During the bottleneck period (from *T*b to *T*e, forward in time), the population size was *N*b. Four statistics, including the segregating sites, Tajima's *D*, nucleotide diversity, and Fu's *F*s, were used to summarize the genetic information in the sequence dataset. We used the standard algorithm in ABCsampler (Wegmann et al. 2010) and the program fastsimcoal (Excoffier and Foll 2011) to simulate samples using the gene information given in Table S1. The mutation rate was set to 1 × 10^−8^ substitutions per site per generation (Li et al. 2010; Sun et al. 2014), and the prior of each parameter was assumed to follow the uniform distribution. We excluded simulated samples with exceptional values because their inclusion would lead to failure when processing them. A total of 1,500,000 samples (500,000 samples for each species and also for pooled data) were simulated. We retained the 5000 simulated samples which were closest to the observed values for each model to calculate marginal density and to generate posterior distributions for *T*b and *T*e with the regression adjustment general linear model.

**Table 2:** Neutrality tests at 13 loci of *Picea asperata* and *P. crassifolia*. Significance was estimated with coalescent simulations under the standard neutral model

Species	Locus	*D*	D*	F*	*H*
*P. asperata*	*4CL*	NA	NA	NA	NA
	*EBS*	1.40366	0.99035	1.33985	−2.67636
	*FT3*	−0.50136	1.07476	0.63631	−0.21234
	*GI*	−0.78037	0.64492	0.22832	0.14623
	*M007D1*	1.50449	1.28112	1.62381	0.37403
	*MOO2*	0.22936	−0.95538	−0.63035	−4.76455
	*PCH*	NA	NA	NA	NA
	*Sb16*	1.63749	1.61185*	1.94473*	−0.48221
	*Sb29*	−1.24090	−3.13200*	−2.93897*	−0.74571
	*Sb62*	2.01848	0.99035	1.58531	−0.88247
	*se1364*	−0.51517	0.46067	0.18747	−1.74545
	*se1390*	2.84105**	1.28112	2.19279**	1.08987
	*xy1420*	NA	NA	NA	NA
	Average	0.65967	0.42478	0.61693	−0.98990
*P. crassifolia*	*4CL*	NA	NA	NA	NA
	*EBS*	1.30445	1.04717	1.35631	−2.52170
	*FT3*	0.42404	0.96298	0.92848	−0.41189
	*GI*	−1.12984	−1.26861	−1.44831	0.04652
	*M007D1*	1.20177	1.36880	1.56878	0.55737
	*MOO2*	1.71182	1.31407	1.75077*	−1.8799
	*PCH*	−0.94180	0.62305	0.14362	−3.73153
	*Sb16*	0.82033	1.55662*	1.53182	−3.03782
	*Sb29*	0.02102	1.19189	0.92605	−0.01658
	*Sb62*	3.16062**	1.04717	2.12439**	0.11695
	*se1364*	−0.76360	0.62305	0.21121	−1.66708
	*se1390*	2.45744*	1.31407	2.07834**	0.27232
	*xy1420*	−0.88170	0.44402	0.03497	0.01581
	Average	0.61538	0.85202	0.93387	−1.02146

*D*, Tajima's D statistic (Tajima 1989); D* and F*, Fu and Li's D* and Fu and Li's F* (Fu and Li 1993); *H*, Fay and Wu's *H* (Zeng et al. 2006).

Significance level: *P <* *0.05; **P <* *0.01. NA, failed to compute due to insufficient variation.

## Results

### Stable morphological difference between two species in a common garden

Needles of the trees that were five or more years old were collected from the upper side of branchlets (five needles per tree) and compared. The differences in leaf apex between species were obvious, and the needles of *P. crassifolia* were always wider than those of *P. asperata*, when comparing needles of the same length (means, 1.44 mm < 1.69 mm, Student's *t*‐test, *P *<* *0.001; Fig. 1). These observations suggested that the morphological differences between these two species are stable and are likely to be caused by genetic changes during their divergent histories.

### Nucleotide diversity and population structure

Genetic diversity was detected at 12 loci (Tables S1, S2). No variation was detected at nuclear locus 4*CL* (Table S2). A total of 71 and 78 polymorphic sites were detected in *P. asperata* and *P. crassifolia*, respectively (Table S1). The silent nucleotide diversities within *P. asperata* and *P. crassifolia* were 0.00334 and 0.00393, respectively. Similarly, the total nucleotide diversity in *P. asperata* (0.00264) was slightly lower than in *P. crassifolia* (0.00304).

**Figure 2:**
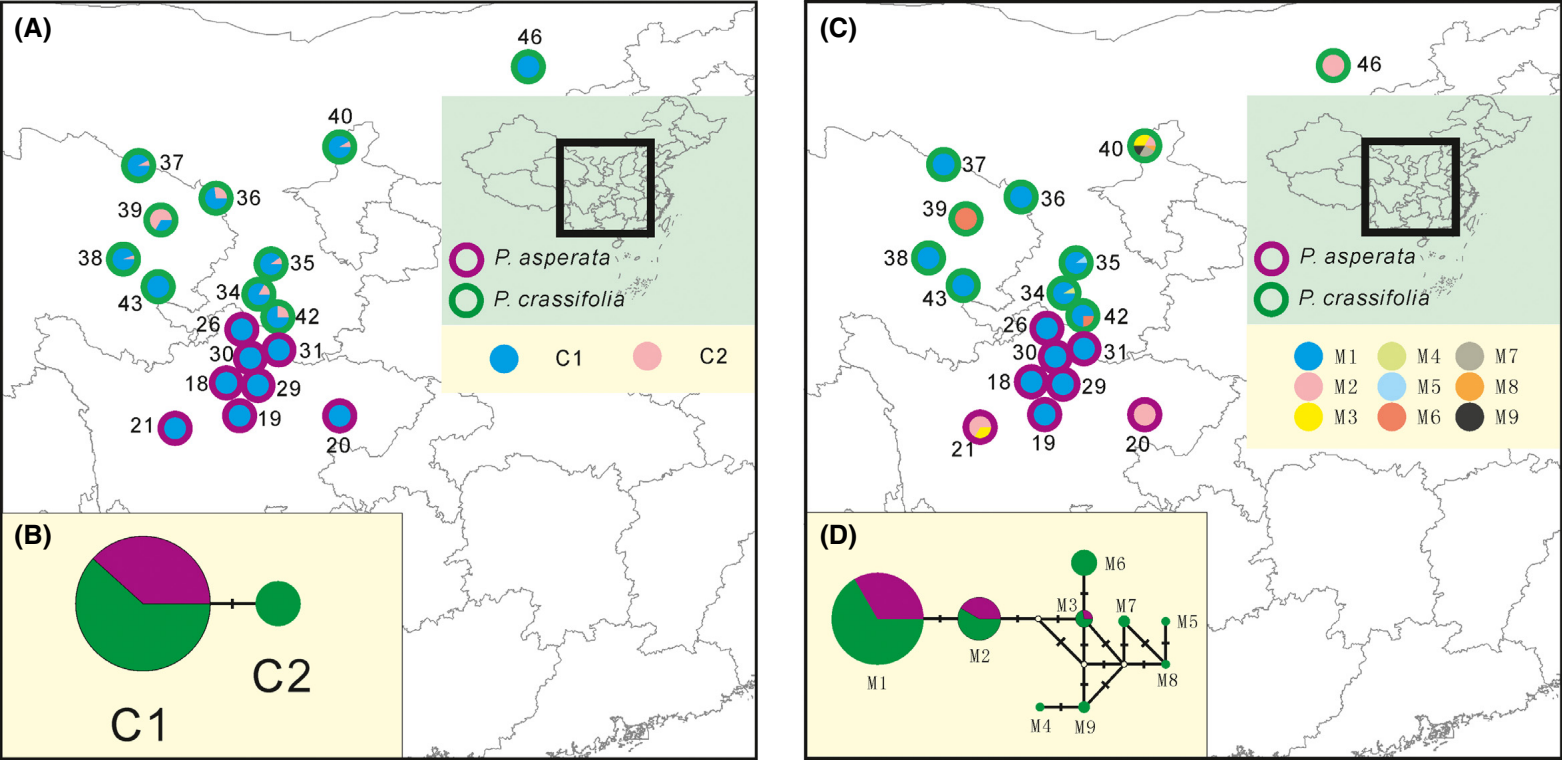
Distributions and networks of chlorotypes (A and B) and mitotypes (C and D) between *P. asperata* and *P. crassifolia*. The circumference colors of circles in (A) and (C) indicate each species. The color‐filled pies in (A) and (C) are proportional to the frequencies of each chlorotype or mitotype in each population. In (B) and (D), each sector of a circle is proportional to the frequencies of each chlorotype or mitotype in each species.

**Figure 3:**
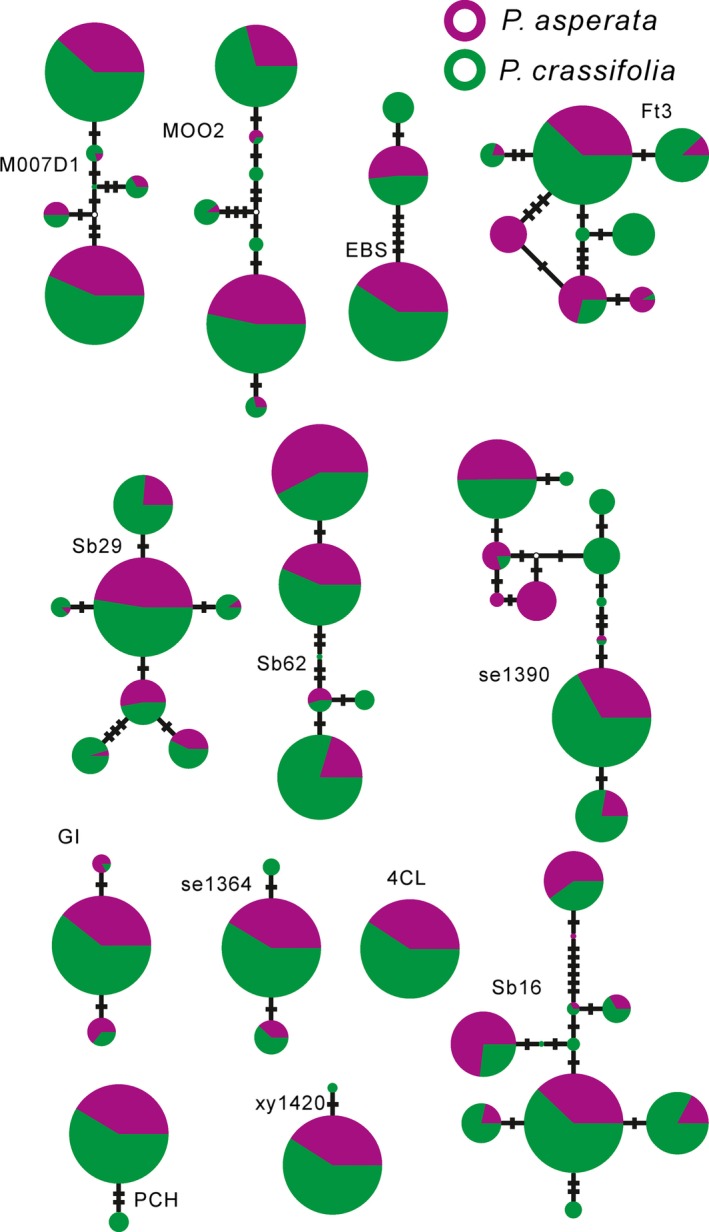
Gene genealogies of the thirteen nuclear loci. Colors in the pie chart indicate the haplotype origin. The size of the pie is proportional to the haplotype frequency found in the two spruce species. Branch lengths longer than one mutation step are marked on each branch.

**Figure 4:**
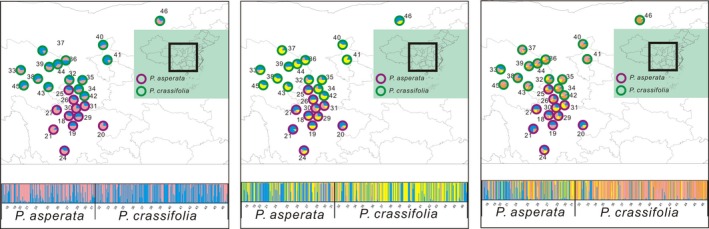
Structure analyses of the two spruce species assuming *K *= 2–4 clusters. Populations are presented as pie charts in which individuals are colored based on mixed membership.

The fixed index *Φ*_ST_ between *P. asperata* and *P. crassifolia* (0.053) was significantly higher than in the random scenario (*P *<* *0.001), indicating clear population differentiation between the species. However, genealogies constructed based on the variations in cpDNA, mtDNA, and at each nuclear locus revealed that *P. asperata* and *P. crassifolia* shared most of their genetic variations (Figs. 2, 3). Like the NETWORK analyses which revealed high levels of sharing allele diversity (Figs. 2, 3), the result of STRUCTURE showed that the most likely number of clusters (*K*) is 2 (Fig. S2). When *K* had a value of 2–4, we found that these two species could not be grouped into distinct clusters (Fig. 4).

### Demographic inference and the parameterized IM model

To examine the demographic dynamics of *P. asperata* and *P. crassifolia*, we performed three tests: Tajima's *D*, Fu and Li's D* and F* tests. Positive average values of *D*, D* and F* were found (Table 2), indicating *P. asperata* and *P. crassifolia* may have experienced recent population bottlenecks.

**Figure 5:**
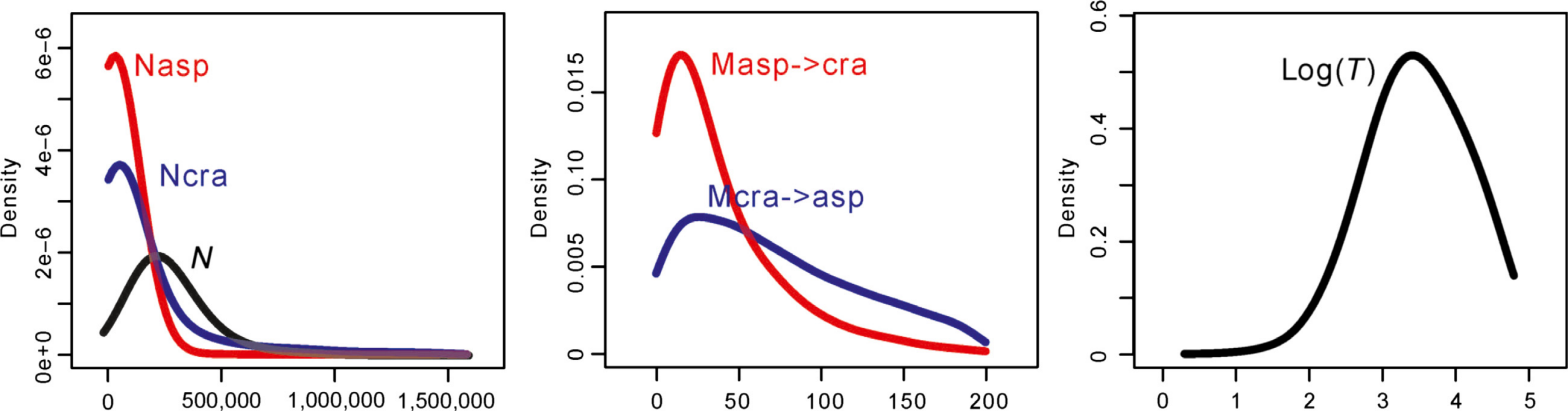
Posterior distribution of parameters within the isolation and migration model based on the sequences from nuclear loci. *N*asp, *N*cra, and *N* indicate the effective population size of *P. asperata*,* P. crassifolia*, and their ancestral lineage, respectively. *M*asp‐>cra denotes the migration rate from *P. asperata* to *P. crassifolia*, and the reverse migration rate is denoted by *M*cra‐>asp. *T* denotes the divergence time between the two species (logarithmically transformed).

We used the ABC approach to estimate the parameters of the IM model based on the sequences at 13 nuclear loci. The estimated current effective population sizes of *P. asperata* and *P. crassifolia* were 3.6 × 10^4^ (95% HPDI: 0.4–19.5 × 10^4^) and 5.2 × 10^4^ (95% HPDI: 0.4–69.1 × 10^4^) individuals, and their ancestral population size (*N*) was 2.4 × 10^5^ (95% HPDI: 0.1–59.3 × 10^4^) individuals (Fig. 5), indicating that population bottlenecks had occurred in their evolutionary histories. The divergence was dated to have occurred 2551 (95% HPDI: 180–56810) generations ago (Table 3), when the population was much smaller than at present and less than 0.02*N. Using a scale of 50 (or 25) years per generation (Heuertz et al. 2006; Ru et al. 2016), their divergence occurred 127.6 (or 63.8) thousand years ago. The migration rate from *P. asperata* to *P. crassifolia* was estimated at 14.14 (95% HPDI: 0–109.1) per generation, and the reverse migration rate from *P. crassifolia* to *P. asperata* was estimated at 26.26 (95% HPDI: 0–169.7) per generation.

**Table 3:** Posterior mode estimates and 95% highest posterior density (HPD) intervals for demographic parameters in the IM model based on the nuclear multilocus nuclear sequence data. *N*asp and *N*cra, current population size of *Picea asperata* and *P. crassifolia*, respectively; *N,* effective population size of the common ancestor; *T*, divergence time between *P. asperata* and *P. crassifolia*;* M*cra‐>asp, the effective migration rate from *P. crassifolia* to *P. asperata*;* M*asp ‐>cra, the effective migration rate from *P. asperata* to *P. crassifolia*

Parameter	*N*asp	*N*cra	*N*	*T* (years)	*T* (generations)	*M*cra‐>asp	*M*asp‐>cra
Mode	35,919	51,887	240,982	127,550	2551	26.26	14.14
HPD 95 lower bound	3981	3981	1000	9000	180	0	0
HPD 95 upper bound	195,606	690,638	592,955	2,840,500	56,810	169.7	109.09

**Figure 6:**
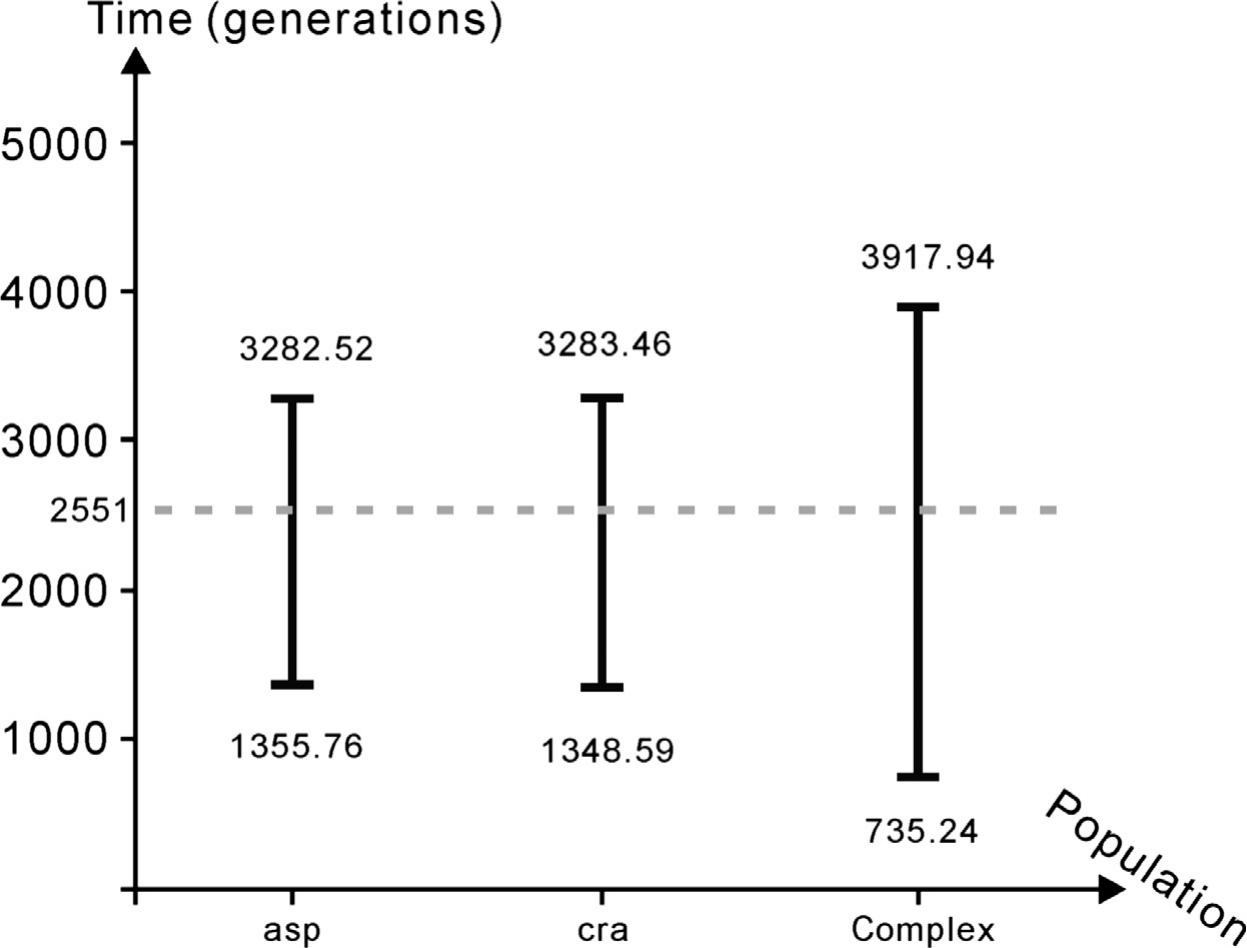
Estimated times of bottlenecks based on the population genetic sequences sampled from *P. asperata* (asp) and *P. crassifolia* (cra). The third bottleneck timing was estimated based on the pooled data (complex). The dotted line represents the divergence time estimated using an ABC approach.

Next, using ABCtoolbox (Wegmann et al. 2010), we estimated the time of the bottleneck based on data from each species and the pooled data. The bottlenecks that these two species experienced occurred ~196,000–37,000 years (Fig. 6, 95% HPDI, a scale of 50 years per generation) ago, and the divergence between *P. asperata* and *P. crassifolia* accompanied with the bottleneck. Thus, the bottleneck likely began earlier than the divergence between *P. asperata* and *P. crassifolia,* and the reduced population size should have restricted gene flow between *P. asperata* and *P. crassifolia* when splitting occurred.

## Discussion

In this study, we integrated two approaches – transplant experiments and population genetic analyses – to examine the phenotypic difference and whether the population demography promoted the initial divergence between two conifer species, *Picea asperata* and *P. crassifolia*. The population size of the ancestral lineage before splitting is larger than the current population sizes of *P. asperata* and *P. crassifolia* (Fig. 5). The interlineage divergence likely occurred following the onset of population shrinkage (Fig. 6), indicating that serious bottlenecks during the late Pleistocene period might have promoted the divergence between *P. asperata* and *P. crassifolia*. The distinct phenotypic differences between *P. asperata* and *P. crassifolia* and extensive genetic sharing indicate that some undetected divergent loci may have contributed to these species' morphological divergences. Our results highlight the importance of Pleistocene climate change in promoting species divergence through serious bottlenecks.

### Population shrinkages in response to climate warming

Silent and total nucleotide diversities for *P. asperata* and *P. crassifolia* are lower than other related spruce species found in the east of the QTP and adjacent regions (Li et al. 2010). For example, the total nucleotide diversities of *P. likiangensis* (0.0093) and *P. wilsonii* (0.0087) are much higher than either *P. crassifolia* (0.00304) or *P. asperata* (0.00264). Recently, *P. likiangensis* and *P. wilsonii* have been shown to have experienced population shrinkages during the late Pleistocene period (Sun et al. 2015). Thus, the lower levels of nucleotide diversity in both *P. asperata* and *P. crassifolia* suggest that these two species may have experienced more severe reductions in their population sizes. This suggestion is also supported by the positive values of Tajima's *D*, Fu and Li's D* and F* at 12 nuclear loci (Table 2). Furthermore, the estimated current population sizes of *P. asperata* and *P. crassifolia* are much smaller than their ancestral population size (Fig. 5 and Table 3). Therefore, we conclude that the population bottlenecks might occur during the evolution of *P. asperata* and *P. crassifolia*.

During the late Pleistocene period, the average temperature in the eastern QTP increased following the retreat of the largest glaciation, which occurred around 800,000 years ago (Shi et al. 1998). However, the low temperature continued until the late Ionian stage, around 300,000–126,000 years ago (Shi et al. 1998). In response to the climate warming, the ranges of cold‐adapted species, such as spruce and yew, contracted, in contrast to the range expansions found for other plants during the inter‐ or postglacial periods (Liu et al. 2013; Sun et al. 2015). In this study, according to the estimate based on the sequences from all nuclear loci, the bottlenecks of *P. asperata* and *P. crassifolia* began 196,000 years ago (3918 generations, Fig. 6). This estimate is similar to the onset of population size reductions (around 200,000 years ago) in the evolutionary histories of *P. likiangensis* and *P. wilsonii* (Sun et al. 2015). Therefore, the climate rewarming during the late Pleistocene period appears to have reduced the ranges and population sizes of many cold‐adapted species distributed in the eastern QTP.

### Divergence between *Picea crassifolia* and *P. asperata*

For spruce species occurring on the QTP and in adjacent regions, most divergences between related species have been found to predate the largest glacial event (Li et al. 2010, 2013, 2015; Zou et al. 2013). A recent divergence (around 127,000 years ago) between *P. asperata* and *P. crassifolia* was revealed here based on the analysis of the IM model (Table 3), indicating that these two species are at the initial stage of recent speciation. The lack of fixed variation (Table S2) also supported this conclusion of recent divergence. Simulations have shown that at such a stage of speciation, there is insufficient time to accumulate genetic differentiation between the newly diverged lineages (Nielsen and Wakeley 2001). Incomplete lineage sorting results in extensive genetic sharing between lineages, as revealed by our STRUCTURE and NETWORK analyses (Figs. 2, 4). An alternative explanation for these shared variations is the gene flow between the two species (Table 3). Primary/second contacts of the distributional ranges of *P. asperata* and *P. crassifolia* after the initial divergence could also have provided opportunities for gene exchange (Fig. S1). *Picea asperata* and *P. crassifolia* bear stable phenotypic differences despite of high level of genetic sharing (Fig. 1), suggesting that the species boundary might be mainly maintained by the divergent selection although the underlying genetic mutations are unknown.

Environmental differences can promote adaptive divergence through selection. Similarly, the reduced gene flow caused by limited dispersal ability and decreased population size can also promote interspecific divergence by accelerating the fixing of different adaptive alleles in the diverging lineages (Räsänen and Hendry 2008). In this study, we found that when *P. asperata* and *P. crassifolia* diverged, their populations were much smaller than their ancestral lineage (Figs. 5, 6). Therefore, we tentatively conclude that the reduced population sizes restricted interspecific gene flow and further promoted divergence between *P. asperata* and *P. crassifolia*, although some interspecific gene flow continued (Table 3).

## Conflict of Interest

None declared.

## Supporting information


**Figure S1.** Locations of each of the sampled populations for the two spruce species studied here, *P. asperata* and *P. crassifolia*.
**Figure S2.** Estimated number of clusters (*K*) obtained with Structure.
**Table S1.** Nucleotide variation at 13 loci in *P. asperata* and *P. crassifolia*.
**Table S2.** The number of segregating sites at 13 loci for *P. asperata* and *P. crassifolia*.
