# Supplementary material for: Late Pleistocene climate change promoted divergence between Picea asperata and P. crassifolia on the Qinghai–Tibet Plateau through recent bottlenecks
Source: Ecol Evol. 2016 Jun 7;6(13):4435–44. doi: 10.1002/ece3.2230 (PMC4930991; doi:10.1002/ece3.2230)
Supplement: Supplementary file 1 — Figure S1. Locations of each of the sampled populations for the two spruce species studied here, P. asperata and P. crassifolia. Figure S2. Estimated number of clusters (K) obtained with Structure. Table S1. Nucleotide variation at 13 loci in P. asperata and P. crassifolia. Table S2. The number of segregating sites at 13 loci for P. asperata and P. crassifolia. [file ECE3-6-4435-s001.docx]

Fig. S1 Locations of each of the sampled populations for the two spruce species studied here, *P. asperata* and *P. crassifolia.* The inset box shows the location of the studied area in a

large-scale map of China. Different colors represent different species.


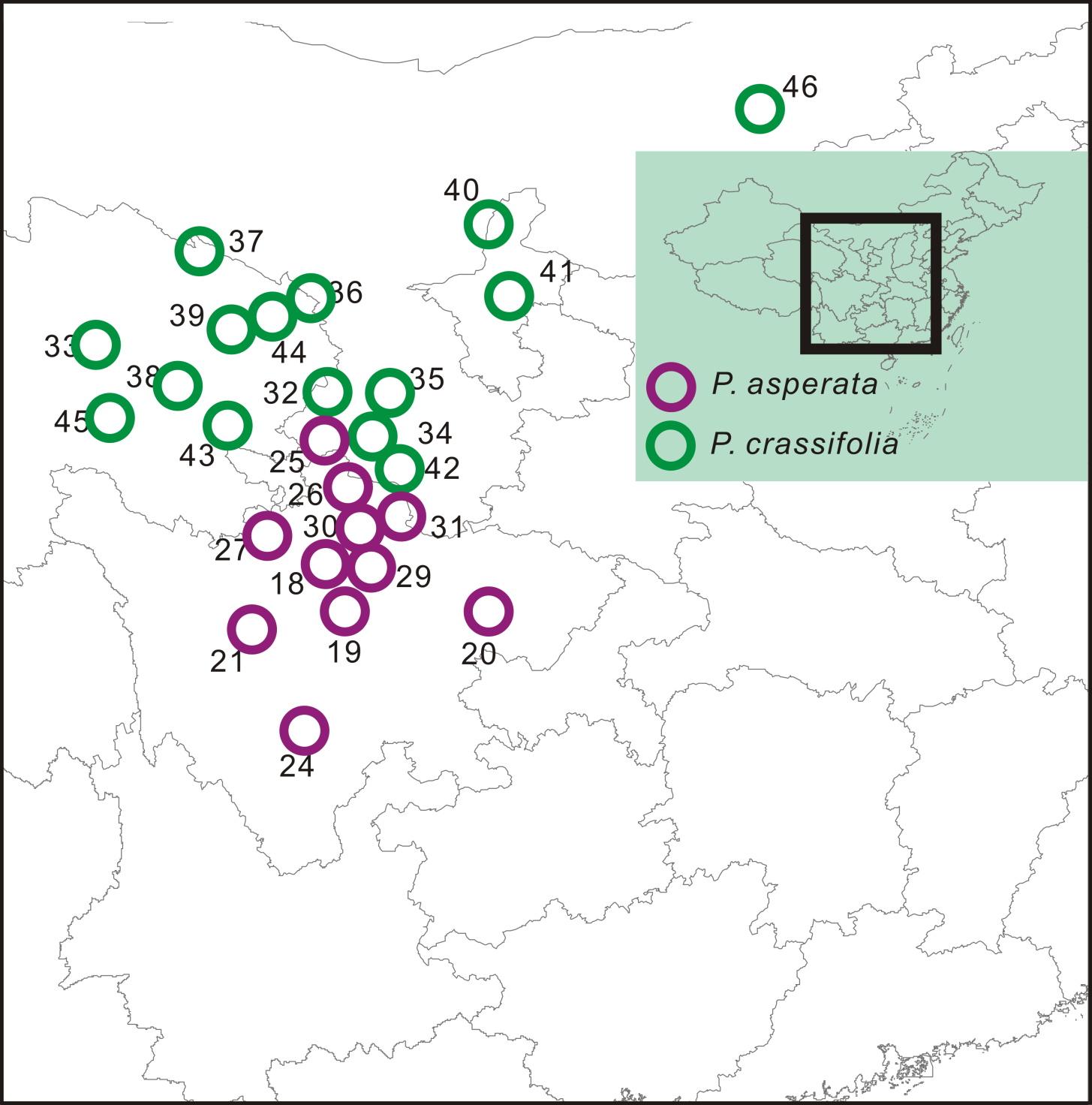


Fig. S2 Estimated number of clusters (*K* ) obtained with Structure. (a) The mean of LnpD with 200 runs considered. (b) ΔK analysis across 10 independent Structure runs (*K* = 1-10), each with 20 repeats, assuming admixture and correlated allele frequencies.


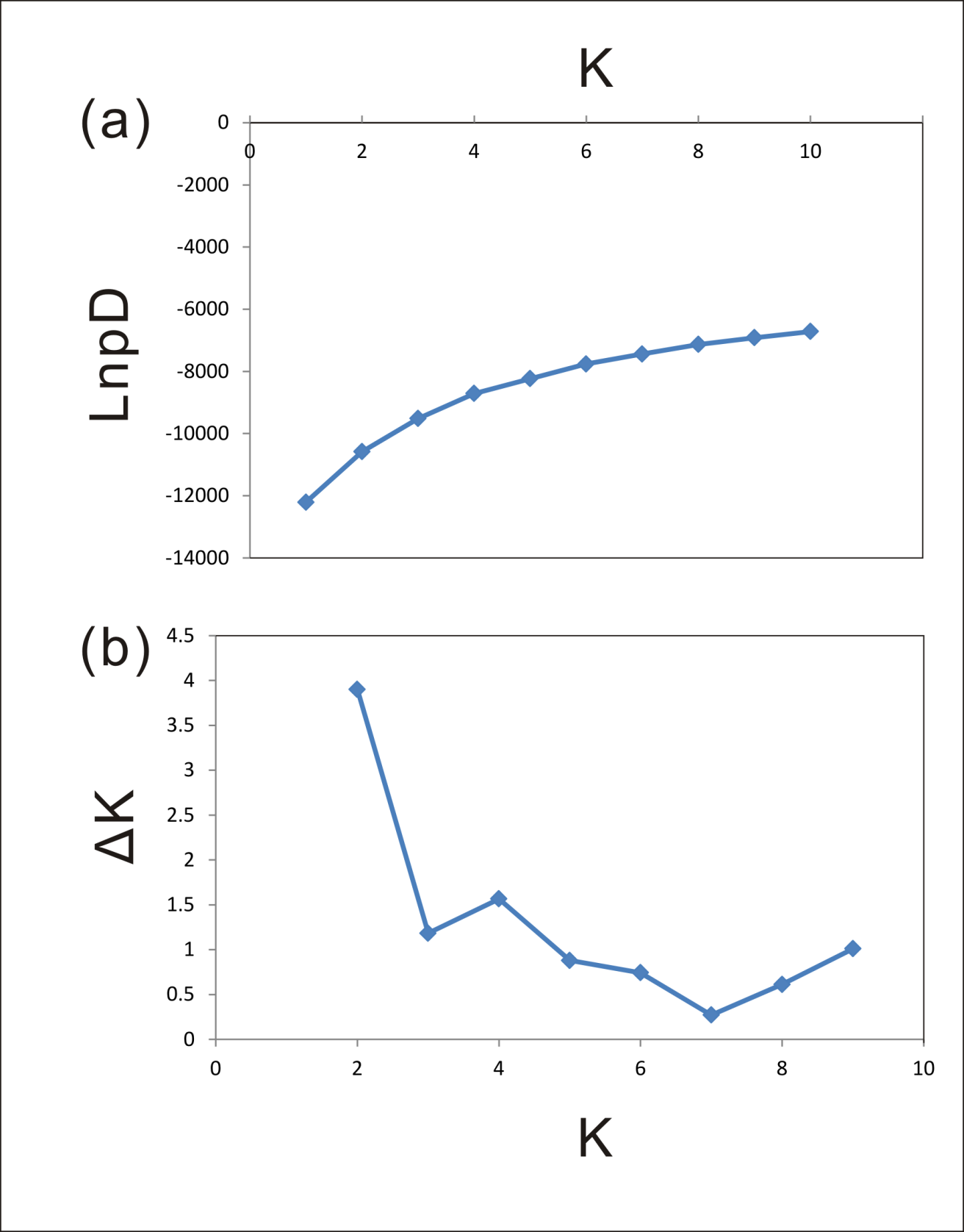


Table S1 Nucleotide variation at 13 loci in *P. asperata* and *P. crassifolia*

| Species | Locus | Total | | | | |  | Nonsynonymous sites | |  | Silent sites | |  |  |
| --- | --- | --- | --- | --- | --- | --- | --- | --- | --- | --- | --- | --- | --- | --- |
|  |  | N | L | S | θ_wt_ | π_t_ |  | θ_wa_ | π_a_ |  | θ_sil_ | π_sil_ |  |  |
|  | *4CL* | 176 | 416 | 0 | 0 | 0 |  | 0 | 0 |  | 0 | 0 |  |  |
|  | *EBS* | 176 | 412 | 5 | 0.00365 | 0.00363 |  | 0 | 0 |  | 0.00246 | 0.00422 |  |  |
|  | *FT3* | 176 | 451 | 6 | 0.00176 | 0.00175 |  | 0.00110 | 0.00080 |  | 0.00301 | 0.00229 |  |  |
|  | *GI* | 176 | 633 | 2 | 0.00024 | 0.00024 |  | 0.00075 | 0.00024 |  | 0.00043 | 0.00024 |  |  |
|  | *M007D1* | 176 | 506 | 9 | 0.00515 | 0.00511 |  | 0.00130 | 0.00033 |  | 0.00513 | 0.01051 |  |  |
|  | *MOO2* | 176 | 425 | 10 | 0.00452 | 0.00449 |  | 0.00107 | 0.00189 |  | 0.00596 | 0.00610 |  |  |
| *P. asperata* | *PCH* | 176 | 558 | 0 | 0 | 0 |  | 0 | 0 |  | 0 | 0 |  |  |
|  | *Sb16* | 176 | 690 | 16 | 0.00662 | 0.00657 |  | 0.00281 | 0.00383 |  | 0.00451 | 0.00762 |  |  |
|  | *Sb29* | 176 | 397 | 8 | 0.00157 | 0.00157 |  | 0.00392 | 0.00196 |  | 0.00205 | 0.00013 |  |  |
|  | *Sb62* | 176 | 430 | 5 | 0.00415 | 0.00413 |  | 0.00146 | 0.00306 |  | 0.00226 | 0.00445 |  |  |
|  | *se1364* | 176 | 428 | 1 | 0.00020 | 0.00020 |  | 0 | 0 |  | 0.00075 | 0.00038 |  |  |
|  | *se1390* | 176 | 528 | 9 | 0.00668 | 0.00662 |  | 0.00223 | 0.00605 |  | 0.00407 | 0.00750 |  |  |
|  | *xy1420* | 176 | 248 | 0 | 0 | 0 |  | 0 | 0 |  | 0 | 0 |  |  |
|  | Average | 176 | 471 | 5.5 | 0.00266 | 0.00264 |  | 0.00113 | 0.00140 |  | 0.00236 | 0.00334 |  |  |
|  | *4CL* | 250 | 416 | 0 | 0 | 0 |  | 0 | 0 |  | 0 | 0 |  |  |
|  | *EBS* | 250 | 412 | 6 | 0.00399 | 0.00397 |  | 0 | 0 |  | 0.00278 | 0.00461 |  |  |
|  | *FT3* | 250 | 451 | 5 | 0.00224 | 0.00223 |  | 0 | 0 |  | 0.00284 | 0.00348 |  |  |
|  | *GI* | 250 | 633 | 2 | 0.00007 | 0.00007 |  | 0.00071 | 0.00003 |  | 0.00041 | 0.00010 |  |  |
|  | *M007D1* | 250 | 506 | 11 | 0.00545 | 0.00541 |  | 0.00183 | 0.00041 |  | 0.00552 | 0.01105 |  |  |
|  | *MOO2* | 250 | 425 | 10 | 0.00683 | 0.00677 |  | 0.00101 | 0.00265 |  | 0.00562 | 0.00931 |  |  |
| *P. crassifolia* | *PCH* | 250 | 558 | 2 | 0.00017 | 0.00017 |  | 0 | 0 |  | 0.00064 | 0.00018 |  |  |
|  | *Sb16* | 250 | 690 | 15 | 0.00479 | 0.00476 |  | 0.00177 | 0.00299 |  | 0.00427 | 0.00544 |  |  |
|  | *Sb29* | 250 | 397 | 8 | 0.00335 | 0.00334 |  | 0.00369 | 0.00377 |  | 0.00193 | 0.00181 |  |  |
|  | *Sb62* | 250 | 430 | 6 | 0.00602 | 0.00597 |  | 0.00137 | 0.00420 |  | 0.00266 | 0.00667 |  |  |
|  | *se1364* | 250 | 428 | 2 | 0.00032 | 0.00032 |  | 0.00083 | 0.00020 |  | 0.00071 | 0.00043 |  |  |
|  | *se1390* | 250 | 528 | 10 | 0.00652 | 0.00646 |  | 0.00210 | 0.00549 |  | 0.00460 | 0.00794 |  |  |
|  | *xy1420* | 250 | 248 | 1 | 0.00006 | 0.00006 |  | 0.00092 | 0.00009 |  | 0 | 0 |  |  |
|  | Average | 250 | 471 | 6 | 0.00306 | 0.00304 |  | 0.00110 | 0.00153 |  | 0.00246 | 0.00393 |  |  |

N, sample size; L, length in base pairs; S, number of segregating sites (number of singletons); π, nucleotide diversity (Nei 1987; Nei and Li, 1979); θ, Watterson’s parameter (Watterson, 1975).

Table S2 The number of segregating sites at 13 loci for *P. asperata* and *P. crassifolia*. S_1_, S_2_ are the number of polymorphic sites unique to the samples of *P. asperata* and *P. crassifolia,* respectively, S_S_ is the number of sites with shared alleles between the two samples, and S_f_ is the number of sites with fixed alleles in either samples.

| Locus | S_1_ | S_2_ | S_S_ | S_F_ |
| --- | --- | --- | --- | --- |
| 4CL | 0 | 0 | 0 | 0 |
| EBS | 5 | 6 | 5 | 0 |
| FT3 | 6 | 5 | 5 | 0 |
| GI | 2 | 2 | 2 | 0 |
| M007D1 | 9 | 11 | 9 | 0 |
| MOO2 | 10 | 10 | 10 | 0 |
| PCH | 0 | 2 | 0 | 0 |
| Sb16 | 16 | 15 | 15 | 0 |
| Sb29 | 8 | 8 | 8 | 0 |
| Sb62 | 5 | 6 | 5 | 0 |
| se1364 | 1 | 2 | 1 | 0 |
| se1390 | 9 | 10 | 9 | 0 |
| xy1420 | 0 | 1 | 0 | 0 |
| Total | 71 | 78 | 69 | 0 |
